# Low Cost Lab on Chip for the Colorimetric Detection of Nitrate in Mineral Water Products

**DOI:** 10.3390/s17102345

**Published:** 2017-10-14

**Authors:** Mohammad F. Khanfar, Wisam Al-Faqheri, Ala’aldeen Al-Halhouli

**Affiliations:** 1 Department of Pharmaceutical and Chemical Engineering, School of Applied Medical Sciences, German Jordanian University, P.O. Box 35247, Amman 11180, Jordan; Mohammad.Khanfar@gju.edu.jo; 2 NanoLab, School of Applied Technical Sciences, German Jordanian University, P.O. Box 35247, Amman 11180, Jordan; Wisam.AlFakhri@gju.edu.jo

**Keywords:** colorimetric, microfluidic, sensor, LOC

## Abstract

The diagnostics of health status and the quality of drinking water are among the most important United Nations sustainable development goals. However, in certain areas, wars and instability have left millions of people setting in refugee camps and dangerous regions where infrastructures are lacking and rapid diagnostics of water quality and medical status are critical. In this work, microfluidic testing chips and photometric setups are developed in cheap and portable way to detect nitrate concentrations in water. The performed test is designed to work according to the Griess procedure. Moreover, to make it simple and usable in areas of low resource settings, commercially available Arduino mega and liquid crystal display (LCD) shield are utilized to process and display results, respectively. For evaluation purposes, different local products of tap water, bottled drinking water, and home-filter treated water samples were tested using the developed setup. A calibration curve with coefficient of determination (R^2^) of 0.98 was obtained when absorbance of the prepared standard solutions was measured as a function of the concentrations. In conclusion, this is the first step towards a compact, portable, and reliable system for nitrate detection in water for point-of-care applications.

## 1. Introduction

The photometric determination of chemical species is one of the key techniques in chemical analysis. If the target species does not absorb radiations in the ultraviolet-visible region, it could be introduced as a limiting reactant in a reaction to produce a colored product. The concentration of the target analyte could be deduced from its absorbance, which follows the Beer-Lambert law over a wide, useful range of concentrations [1,2,3].

Nitrate is an anion of significant interest, since it could be hazardous to public health if accumulated in human body in concentrations higher than 500 ppm. Through a series of chemical reactions in the body, nitrate is reduced to nitrous acid, which oxidizes the ferrous ion of the hemoglobin to the 3+ oxidation state, converting the hemoglobin to methemoglobin (brown) which does not transport oxygen as efficiently. This causes a disease known as methemoglobinemia or “blue baby syndrome” that is accompanied with a fast heart rate and shortness of breath, and could result in death [4,5,6].

The photometric detection of nitrate is based on the Griess test. In brief, nitrate is reduced to nitrite by means of cadmium in an acidic solution. Sulfanilamide is added to the nitrite to produce a cation known as diazonium salt. The last step in the test procedure is coupling the salt with N-alpha-naphthyl-ethylenediamine to yield the azo dye, which has a pink color. The intensity of the pink color is correlated to the original nitrate concentration. Photometric (or colorimetric) measurements are based on the Beer-Lambert law; in brief, the absorbing ingredient, also known as chromophore, absorbs portion of the radiant energy emitted by a light source. As a consequence, the transmitted light is attenuated to an extent that is directly proportional to the amount of the absorbing species. The key parameters, the absorbance (A) and the concentration (c), are connected via the relationship: A=ε·b·c, where ε is the absorptivity coefficient and b is the distance the light passes across the analyte solution [7,8,9,10].

Colorimetric determination of chemicals could be more versatile if conducted on a smaller scale, rather than an industrial scale. For field measurements, it is practical to use portable measuring devices that could provide the operator with a rapid quantitative determination of the target compounds. Miniaturization of the lab-scale equipment and measuring devices could be achieved through the utilization of lab on a chip technology, where mixing processes take place in grooves and/or channels patterned on paper or plastic templates and the desirable quantitative analysis of the target species is performed with the assistance of compact electronic circuits that function in a manner similar to that of the lab-scale measuring instruments [11,12,13]. In addition, miniaturizing detection systems provides the advantages of fast analysis, parallelization, low cost, portability, minute reagent consumption, the possibility of running the system by non-trained public workers, and the option of use in areas of low resource settings [14].

The detection of nitrate concentration at the micro molar level has been investigated by different research groups. Optimization of the detection conditions include adjusting the pH of the analyte, its flow rate, the utilized substrates, and the performance of the light-emitting diode and the photodiode array systems used for the detection purposes. The fabricated setups have been mainly used for the determination of nitrate and nitrite concentrations in aqueous systems such as sea water and wastewater for environmental monitoring purposes [15,16,17,18,19,20].

In this work, a home-made photometric miniaturized detection system was fabricated and its performance toward the detection of nitrate in mineral water products supplied locally was examined and utilized for the colorimetric detection of nitrate in local drinking waters. The fabricated system is simple in design and operation, and its reported results are reproducible and precise.

## 2. Microfluidic Platform Design and Fabrication

**Figure 1:**
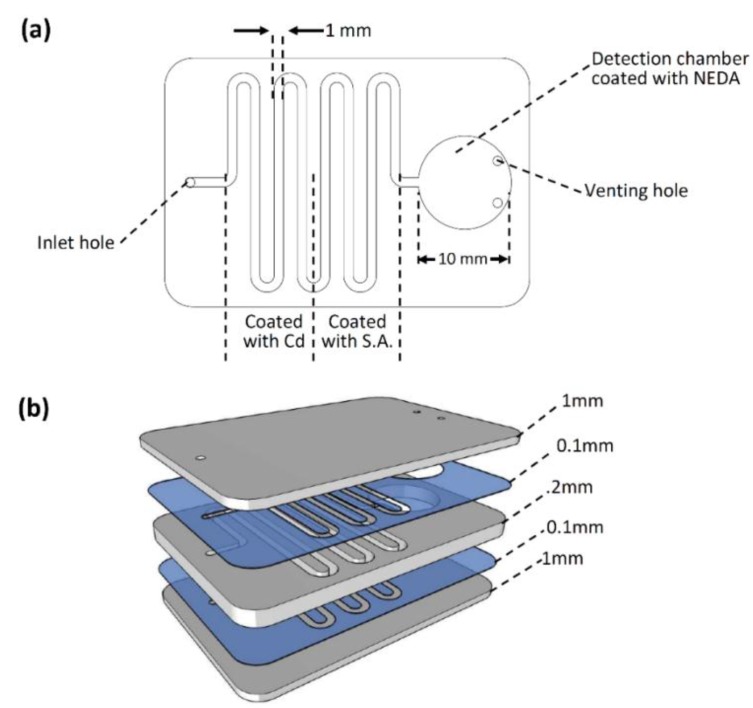
Microfluidic chip for nitrate detection. (**a**) Chip design where a 1 mm × 2 mm spiral channel with one inlet is connected to a final detection chamber; (**b**) microfluidic chip layers.

For this project, a microfluidic chip with a straight-forward design was designed and fabricated. As can be seen in Figure 1a, the microfluidic chip consists of a curved channel with a 1-mm width and 2-mm depth. The spiral channel has a single inlet hole for sample load and pumping. The channel is connected to a 10-mm diameter detection chamber where the final chemical reaction and detection will be performed. The detection chamber has two venting holes to allow air movement (in and out) during fluid flow process.

The final microfluidic chip design was fabricated on polymethyl methacrylate (PMMA) plastic (Moden Glas, Bangkok, Thailand). The fabricated chip has three PMMA layers: two 1-mm layers on the top and bottom of the chip, as well as a 2-mm layer in the middle (see Figure 1b). The main microfluidic features (the channel and the detection chamber) were cut into the middle 2-mm PMMA layers. The inlet hole and two venting holes were cut into the top layer, while the bottom PMMA layer has no features (implemented as a cover only). All the microfluidic features were introduced into the PMMA layers using Bodor CO_2_ laser cutter (Bodor, Shandong, China). Afterwards, the machined PMMA layers pass through different steps of cleaning, washing, and drying before the bonding process starts. The cleaned PMMA layers were aligned and bonded together using two pressure-sensitive adhesive (PSA) layers (FLEXcon, Spencer, MA, USA). A 100-µm, texture- and color-free PSA is implemented in this work to avoid any signal and/or absorbance interruption.

### 2.1. Detection Setup

**Figure 2:**
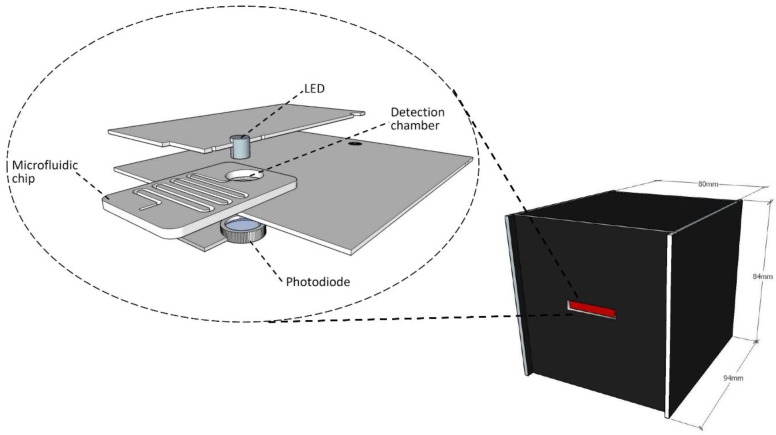
Colorimetric detection setup (on the right) external view of the detection setup (on the left) internal view, top layer with LED, bottom layer with photodiode, and middle layer is the holder for microfluidic chip

In this work, a simple and cost-effective colorimetric detection setup that can read the final reaction results directly from the detection chamber has been designed and developed. The developed detection setup can facilitate the main goal of the development of a simple, portable, on-site, and cost-effective method for the detection of chemical compounds. Figure 2 shows a 3D view of the proposed detection setup. The main setup is located inside a black semi-cubic box that is 80 mm, 84 mm, and 90 mm in dimension. This black cover has a slot in the front wall for the microfluidic chip to slide in for the final result-reading process. The box is made of 3-mm PMMA sheets and painted black. The inside view shows that the used detection setup consists of three layers: a top layer with a green LED (light-emitting diode) (Farnell, Aschheim, Germany) emits light in the 520–530 nm wavelength range through the detection chamber. The bottom layer is made with a photodiode (Farnell, Aschheim, Germany) which is aligned directly under the LED for light observation and analysis. Finally, the middle layer is made of a specific design to hold and align the detection chamber exactly between the LED and photodiode. For signal read-out and display, Arduino mega, along with LCD shield were utilized. The Arduino was also used as a power source for the LED and the photodiode. For each test, the utilized Arduino takes the voltage signal of the photodiode and displays it on the LCD screen. It was also programmed to display the average of 100 readings each 5 s. Elevation of the nitrate concentration decreases the amount of light transmitted to the photodiode and, as a consequence, the corresponding electrical signal-voltage in this case—decreases. With that, the absorbance (or the transmittance) is correlated to the measured nitrate concentration.

### 2.2. Chemicals Preparation

Cadmium powder, potassium nitrate, and dihydrogen sodium phosphate were purchased from Sigma Aldrich, St. Louis, MO, USA. Phosphoric acid was provided by Riedel de haen (now Honeywell-Riedel de Haen, Morristown, NJ, USA). Benzensulfanylamide (S.A.) was purchased from Applichem GmbH, Darmstadt, Germany, and N-1-naphthylethylenediamin dihydrochloride (NEDA) from Carlo Erba reagents, Peypin, France. Potassium hydroxide was ordered from S.D. Fine Chem Limited, Mumbai, India.

All of the tested solutions were prepared using HPLC grade water obtained from UltraMax 372 Yonglin Water Purification System, Anyang, Korea. Phosphate pH 2.0 buffer solution was employed as the working solution; the solution was prepared by mixing appropriate amounts of dihydrogen sodium phosphate and phosphoric acid. The pH of the prepared solution was adjusted using 0.1 M NaOH aqueous solution.

A 0.5 mM solution of potassium nitrate was prepared and passed through a titration pipette with the cadmium powder located at the bottom of the pipette atop a cotton bed. The reduced nitrate was then mixed with 1.0 mM buffer solutions of S.A. and NEDA to produce the desired standard pink solution. The prepared solution was diluted serially to make standard solutions of lower concentrations. Absorbance of the prepared solutions was measured using the fabricated detection system.

For the detection of the nitrate content in water samples, suspensions of the three ingredients were immobilized on the regions shown in Figure 1 using a micropipette. Masses of the ingredients were selected so that they did exist in excess amounts when compared to the target analyte; the nitrate and the three ingredients, Cd, S.A., and NEDA, were immobilized on the walls of the microfluidic channel as aqueous solutions, then dried at 45 °C for 1 h. The chips were then sealed and became ready for the desired detection procedure.

### 2.3. Operational Concept

**Figure 3:**
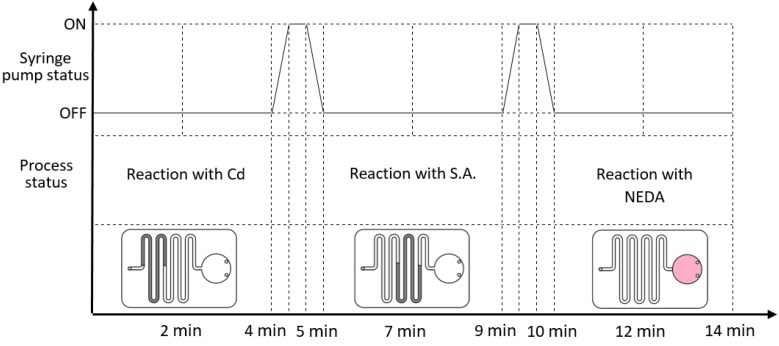
Experimental steps (top part) syringe pump status over time (bottom part) sample position inside the microfluidic chip over time.

The novel microfluidic design which was discussed earlier eliminates the need for any valving or gating elements to perform the chemical reaction. Instead, the design simply requires a pumping method (using syringe pump in our case) to drive the sample through the microfluidic channel while accurately controlling the flow rate. Figure 3 shows how the experimental process works step-by-step. The upper part of the graph presents the syringe pump status over time as it changes from OFF (no liquid flow) to ON (pump the liquid to the next part of the chip). Under each pump status, the process status and liquid position is presented. For the first time cycle (0 min to 4 min), the syringe pump is OFF and the sample is located within the first part of the chip, which is coated with Cd. Afterward, syringe pump is activated for 1 min to drive the sample from Cd-coated part to S.A.-coated part. It can be noted that we implemented a low flow rate to allow for accurate liquid flow control without the need for the integration of valving mechanism. At 5 min, the pump is deactivated and liquid is allowed to react with the S.A. coating for 4 min. At 9 min, the pump is activated again to drive the liquid to its final destination (the detection chamber). In the detection chamber, the liquid reacts with the NEDA coating for 4 min, where liquid color turns pink. At the end of the reaction time, the pink color intensity presents the concentration of nitrate compounds in the processed sample. The microfluidic chip is then slid into the detection setup for the final result reading. The results will be displayed and saved on a PC dedicated for this process.

## 3. Results and Discussion

### 3.1. The Calibration Curve

The main objective of this part is to establish a calibration curve that correlates the produced dye absorbance to the nitrate concentration in parts per million (ppm). As shown in Table 1, the extent of light absorption increases as the nitrate concentration (standard solutions) increases.

**Figure 4:**
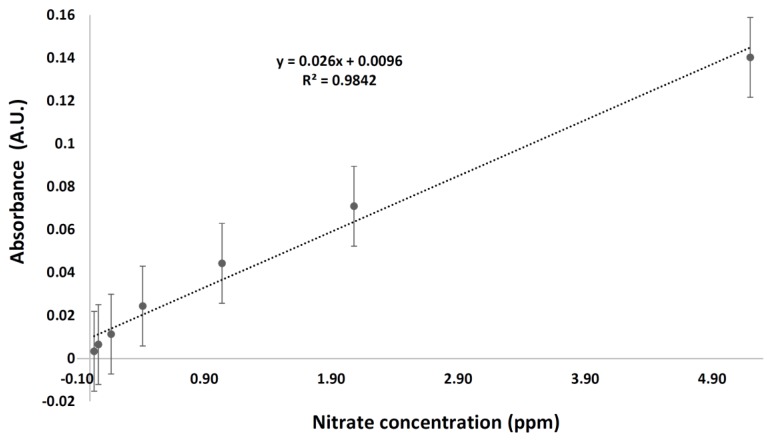
Calibration curve for the prepared standard solutions.

Absorbance of the standard solutions was measured in six repeats using the phosphate buffer solution as the blank and the fabricated system as mentioned earlier. The reported results are shown in Figure 4.

The results were obtained based on the 535 nm absorbance maxima of the azo dye. As shown in Figure 4, the reported R^2^ value is equal to 0.9842. The limit of detection (LOD) and limit of quantitation (LOQ) were found to be 0.0782 and 0.237 ppm, respectively. The LOD is estimated to be 3 σ/m, while the LOQ is calculated as 10σ/m where σ is the standard deviation of the lowest measured concentration (measured six times) and m is the slope of the calibration curve. These values indicate that the performed work needs further improvement when compared to the previously reported value of 0.0016 mg/L [21]. Issues related to quality of the materials used for the chip fabrication, intensity of the employed incident light, and sensitivity of the used detector must be considered in order to optimize the experimental conditions.

### 3.2. The Water Samples

**Figure 5:**
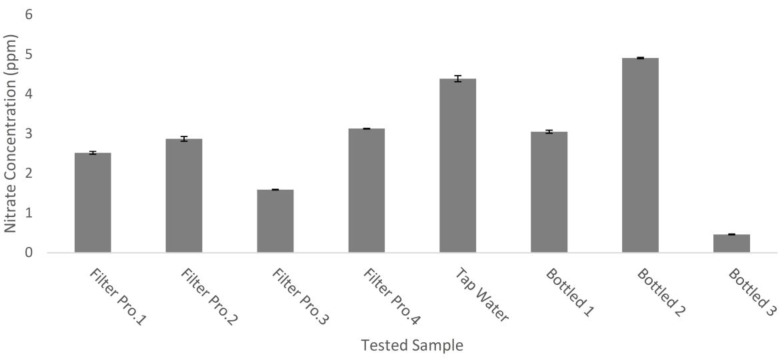
Total nitrate concentration in selected water samples.

In this work, the nitrate content in eight water samples of different origins was measured using the fabricated system. The samples were tap water, bottled drinking water, and home-filter treated water. With the utilization of the fabricated system, the total nitrate content (nitrate and nitrite) was quantified and the corresponding results are listed in Figure 5. In the tested samples, the nitrate content was less than the maximum concentration limit (MCL) of 10 ppm (or 1.176 × 10^−4^ M).

The nitrate content in each sample was measured six times, and the listed results present the average nitrate content in the water samples. As shown in Figure 5, the nitrate content is below the MCL value. Unexpectedly, the maximum reported nitrate content is that of a bottled water sample (Bottled 2), which is even higher than that of the tap water. That reported, and unexpected, value could be attributed to missing key step during course of the purification, which is the anions removal process, allowing nitrate along with other anions to exist as species dissolved in the aqueous matrix with relatively high concentrations. The home-filter treated waters demonstrated moderate nitrate concentrations. The obtained results are of acceptable credibility, since the lowest detected nitrate concentration (Bottled 3) is almost twice as high as the limit of quantification.

The reported results point to the total nitrate concentrations, including nitrate (NO^3−^) and nitrite (NO^2−^). The nitrite content could be estimated if the experiments were repeated without the utilization of cadmium, which is responsible for the nitrate to nitrite reduction. The detection conditions could be improved with the utilization of a glass light transparent cover that transmits the incident radiations more efficiently than the PMMA used in this work; with that, adherence to Beer’s law could be expected in a manner more linear than that shown in Figure 4 (i.e., at R^2^ = 0.9842).

Compared to the previously reported works, our developed setup is simple in design and operation, and its reported results are reproducible and precise. In term of fabrication, the developed microfluidic chip can be fabricated very easily with a basic milling machine or laser cutter with a doable dimension of microfluidic channel (one that is not very small and does not require highly accurate machining). The developed chip is cheap and disposable, without any integrated electronic components such as LED or fiber optics [15,17]. Finally, the developed setup does not need bulky laboratory equipment and/or components such as a controlling PC, data processors, a bulky AC or DC power source (a battery is enough), or a micro-valve for flow control. Even sample pumping can be perfumed manually with a normal pipette or syringe. This makes our setup applicable in low-resource and extreme point-of-care areas.

## 4. Conclusions

In this work, microfluidic testing chips and a colorimetric setup were developed in a cheap and portable way to detect nitrate concentrations in water. The performed test was developed to work according to the Griess procedure. The microfluidic chip was designed to have a long-coated channel fabricated using PMMA layers of different thicknesses. On the other hand, the colorimetric setup mainly consisted of an LED light source, photodiode, Arduino mega, and LCD shield. The developed setup was evaluated using different water samples including bottled water products. The concept of colorimetric detection at the micro level and its viability have been proven, and a useful system for the detection of nitrate in local mineral water products has been fabricated. This chip is easy to fabricate and use, disposable, cheap, and can be operated by non-trained personnel. However, much still needs to be done to improve the detection conditions. In the near future, an effort will be paid to enhance the sensitivity of the spectroscopic detector, thus improving the detection performance of the fabricated setup.

## Figures and Tables

**Table 1:** Dependence of absorbance of the produced dye on the concentration of the standard nitrate solutions.

Standard Solution	Concentration in ppm	Voltage in mV (N = 6)	Absorbance
Blank	0	2845.386667	0.0000
Solution 1	0.033271	2823.436667	0.003363293
Solution 2	0.066541	2803.05	0.006510498
Solution 3	0.166353	2772.118333	0.011329578
Solution 4	0.415882	2689.8	0.024421354
Solution 5	1.039704	2569.236667	0.044337232
Solution 6	2.079408	2416.596667	0.070937171
Solution 7	5.198519	2060.125	0.140247771
